# Whole-Body Cryostimulation in Multiple Sclerosis: A Scoping Review

**DOI:** 10.3390/jcm13072003

**Published:** 2024-03-29

**Authors:** Angelo Alito, Jacopo Maria Fontana, Eleonora Franzini Tibaldeo, Federica Verme, Paolo Piterà, Elzbieta Miller, Riccardo Cremascoli, Andrea Brioschi, Paolo Capodaglio

**Affiliations:** 1 Department of Biomedical, Dental Sciences and Morphological and Functional Images, University of Messina, 98125 Messina, Italy; alitoa@unime.it; 2 IRCCS, Istituto Auxologico Italiano, Orthopedic Rehabilitation Unit, Research Laboratory in Biomechanics and Rehabilitation, San Giuseppe Hospital, Piancavallo, 28921 Verbania, Italy; j.fontana@auxologico.it (J.M.F.); p.capodaglio@auxologico.it (P.C.); 3 Department of Surgical Sciences, Physical and Rehabilitation Medicine, University of Torino, 10121 Torino, Italy; elefratiba@gmail.com; 4 Department of Clinical and Biological Sciences, University of Turin, 10043 Torino, Italy; p.pitera@auxologico.it; 5 Department of Neurological Rehabilitation, Medical University of Lodz, Milionowa 14, 93-113 Lodz, Poland; elzbieta.dorota.miller@umed.lodz.pl; 6 IRCCS, Istituto Auxologico Italiano, Unit of Neurology and Neurorehabilitation, San Giuseppe Hospital, Piancavallo, 28921 Verbania, Italy; r.cremascoli@auxologico.it (R.C.); a.brioschi@auxologico.it (A.B.)

**Keywords:** cold therapy, cryostimulation, multiple sclerosis, neurological disorders, rehabilitation, whole-body cryostimulation

## Abstract

Multiple sclerosis (MS) is the most common cause of non-traumatic long-term disability in young adults. Whole-body cryostimulation (WBC) is a cold-based physical therapy known to induce physiological exercise-mimicking changes in the cardiovascular, neuromuscular, immune, and endocrine systems and to influence functional and psychological parameters by exposing the human body to cryogenic temperatures (≤−110 °C) for 2–3 min. The purpose of this scoping review is to present an overall view on the potential role of WBC as an adjuvant therapy in the treatment of MS. PubMed, ScienceDirect, Embase, and Web of Science were searched up to 30 November 2023, and a total of 13 articles were included. WBC may have beneficial antioxidant effects as a short-term adjuvant treatment in MS. There were no significant changes in antioxidant enzymes, nitric oxide levels, metalloproteinase levels, blood counts, rheology, and biochemistry. WBC can lead to a reduction in fatigue and an improvement in functional status, with a significant effect on both mental and physical well-being. There were no reported adverse effects. The results suggest that WBC may complement therapeutic options for patients with MS, as the effects of cryogenic cold stimulation have been shown to activate antioxidant processes and improve functional status, mood, anxiety, and fatigue.

## 1. Introduction

Multiple Sclerosis (MS) is a chronic inflammatory and neurodegenerative disease characterised by an indeterminable clinical evolution, with high incidence and prevalence rates in young adults leading to early disability [1,2,3]. Although the aetiology is not fully understood, MS is generally classified as an inflammatory disease with an autoimmune response directed against the central nervous system (CNS) [4,5]. MS has considerable variability among patients and its clinical course could be influenced by multiple factors, including gender, age and symptoms and signs at onset [6,7].

Three main patterns could be described in the clinical evolution of MS: relapsing–remitting MS (RRMS), secondary progressive MS (SPMS), and primary progressive MS (PPMS) [8,9,10]. RRMS is the most frequent type and is characterised by periods of worsening symptoms followed by complete or partial recovery, while in the other two patterns the progressive worsening of neurological impairment could be present after a period where there is a relapsing–remitting phase (SPMS) or from the beginning without exacerbations (PPMS) [11,12].

Oxidative stress is the hallmark of neuroinflammation and has been implicated as one of the factors involved in the pathogenesis of MS, particularly because of the participation of reactive oxygen species (ROS) in the demyelinating processes [13,14]. Indeed, people affected by MS in fact show high levels of haematological oxidative stress indicators [15,16], as well as the presence of ROS-dependent DNA damage associated with neurodegeneration [17,18].

Focal CNS lesions are the most specific pathological alterations in MS and are typically defined by different evolutionary phases, characterised by early inflammatory and demyelinating aspects, followed by a progression to overlapping reparative (remyelination) and neurodegenerative phases [19,20]. The signs and symptoms of MS can manifest as a consequence of focal lesions (e.g., focal motor or sensory deficit) or can be the expression of widespread damage as a result of the sum of multiple focal lesions or of the neurodegenerative process (e.g., cognitive impairment, ataxia, spasticity, fatigue, and pain) [1,21,22].

Fatigue occurs in 70–80% of patients and represents one of the most frequent and disabling symptoms at all stages of the disease, affecting social, physical, and occupational well-being [23,24]. Some symptoms may vary depending on environmental conditions such as temperature and humidity, and can have a significant impact on daily functioning [25]. A significant amount (60–80%) of patients experience heat intolerance, a temporary exacerbation of symptoms as body temperature rises [25,26]. The Expanded Disability Status Scale (EDSS) is the most common evaluation disability scale and is used to monitor disease changes over time, but it is not able to quantify nonspecific clinical aspects such as fatigue, pain, and spasticity [27,28].

The treatment for MS can be divided into three categories: therapy for the acute relapsing phase (e.g., steroids); immunomodulation to reduce the biological activity of the disease, the rate of relapses, and the accumulation of lesions and to slow progression, such as disease-modifying therapies (DMTs); and symptomatic treatment, targeting, for example, depression, pain, spasticity, sexual dysfunction, insomnia, and bladder and bowel dysfunction [29,30,31].

Non-pharmacological strategies focus mainly on relieving symptoms and reducing disability and are based on physiotherapy, occupational therapy, speech and swallowing therapy, cognitive rehabilitation, and psychological support [32,33,34].

Numerous studies have shown that exercise provides several benefits, both in motor or psychological functions, having an impact on fatigue and cardiorespiratory fitness, and it also can induce neuroprotective mechanisms that reduce long-term disability [35].

Unfortunately, the clinical disability in people with MS does not always allow for sufficient levels of exercise, so the research and development of alternative and complementary approaches to fatigue and MS symptom relief appears to be of great importance [36,37].

Whole-body cryostimulation (WBC) is an emerging cold-based physical treatment known to induce physiological exercise-mimicking changes in the cardiovascular, neuromuscular, immune, and endocrine systems [38] that are beneficial and effective in maintaining or restoring homeostasis [39,40].

WBC is best known for its use in sports medicine after exercise (training and/or competition) to alleviate exercise-associated muscle soreness; to reduce exercise-related pro-inflammatory reactions, injury, fatigue, and inflammation; and to improve post-exercise muscle recovery [38]. In addition, a rapidly growing body of work suggests that WBC could play a promising role as an adjuvant therapy in the rehabilitation of diverse conditions [39,41,42,43]. In fact, WBC is currently being used to relieve the symptoms of more complex conditions such as depression, anxiety, and sleep disorders, but also rheumatoid arthritis, fibromyalgia, ankylosing spondylitis, post-COVID syndrome, obesity, and, of course, multiple sclerosis [44].

The treatment involves repeated exposure to cryogenic temperatures (≤−110 °C) for 2–3 min, generally in 10–20 sessions/cycle, while wearing light clothing (bathing suit, cap, gloves, socks, shoes, and surgical face mask) [44,45]. The effects of cold stimulation have been shown to be beneficial on several physiological (i.e., haematological, metabolic, energetic, endocrinological, skeletal, muscular, and inflammatory), functional (post-exercise and post-traumatic recovery, pain, and physical performance), and psychological (anxiety, depression, and well-being) parameters [38,46].

Several research groups have investigated the impact of WBC on patients affected by MS, aiming to establish its potential beneficial effects, such as improving overall functionality (strength, coordination, and cognitive abilities) and reducing spasticity, fatigue, and pain [47,48].

The aim of this scoping review is to present a summary of the latest research findings on the potential role of WBC as an adjuvant therapy in MS.

## 2. Materials and Methods

### 2.1. Protocol and Registration

This review was structured around the research question “What are the effects to date of the use of WBC in patients with MS?” and was conducted according to the standards of the Preferred Reporting Items for Systematic reviews and Meta-Analyses extension for Scoping Reviews (PRISMA-ScR) [49]. Table 1 shows the criteria for the PICOS components (population, intervention, comparison, outcome, and study design) of this review.

### 2.2. Search Processing

Pubmed, ScienceDirect, Embase, and Web of Science were screened to identify the articles related to the research topic up to 30 November 2023. The search method was developed by combining words which deal with the effects of WBC on patients affected by MS. The search strategy is provided in Table 2.

### 2.3. Inclusion and Exclusion Criteria

The inclusion criteria were (a) studies, (b) in English, and (c) on the use of WBC in patients diagnosed with MS. The exclusion criteria were grey literature data, review articles, animal studies, and articles without the full-text available.

### 2.4. Selection of Sources of Evidence

**Figure 1:**
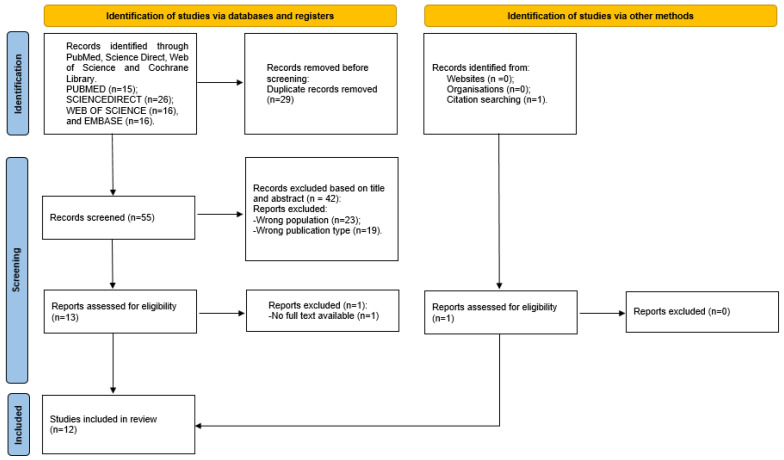
Flowchart summarising the selection process of papers, with the preferred reporting items for systematic reviews and meta-analyses.

Three reviewers (A.A., E.F.T., and F.V.) searched the database to extrapolate the studies, according to the selection criteria. The publications retrieved from the databases were imported into the Rayyan online software (Intelligent Systematic Review) [50] to facilitate the simultaneous evaluation of studies by all researchers. The software was then used to identify and remove any duplicate articles. The reviewers independently analysed the titles and abstracts of the articles according to the eligibility criteria. An evaluation of relevant references was also undertaken for the collection of additional articles. All articles that met the inclusion criteria were preselected for a full-text reading. The selected article data were extracted from the included papers according to relevance. Any disagreement was solved by consensus. The PRISMA flowchart is represented in Figure 1 [51].

### 2.5. Data Extraction

Data extraction was then independently completed by the other two authors (P.C. and P.P.), who also contributed to the analysis process. The accurateness of these data was confirmed by another author (J.M.F.). Disagreements were resolved by consensus. The relevant data were then entered into a database with the agreement of the observers on (1) the study design, (2) the sample size and characteristics, (3) the WBC application, (4) the outcome measures, (5) the time points of the evaluations, and (6) the main results. The authors took into account whether the studies showed adverse effects.

### 2.6. Synthesis of Results

The data were summarised by outcome, with reference to the blood test results, the performance tests, and psychological aspects.

## 3. Results

### 3.1. Study Selection

The search in the PubMed, ScienceDirect, Embase, and Web of Science databases yielded a total of 84 potentially relevant publications. In total, 29 duplicates were excluded from the search prior to screening. After reading the title and abstract, 42 articles were discarded for the following reasons: the wrong target population (*n* = 23) and the wrong study type (i.e., book chapter, conference abstract, poster, and review article) (*n* = 19). Within the remaining thirteen articles, a total of twelve studies were retrieved and one study was not retrieved. The remaining 12 articles were read in full. All were suitable for data extraction (Figure 1). A manual search of the reference lists yielded one article. Finally, 13 articles were included in this review. An overview of the included studies and the extracted data is given in Table 3.

### 3.2. Study Features

The pooled sample of these studies was a total of 1174 patients. Among the twelve studies included, one was a randomised controlled trial (RCT), ten were prospective clinical trials, one was an observational study, and one was a case-control study. Table 1 provides an overview of the included studies and the results.

### 3.3. WBC Application Modality

There was no standardisation of the WBC protocols used in the included studies. The studies presented different types of cryochambers (e.g., Wroclaw, KR2005N), and sometimes this information was missing. The differences were also in terms of the number of sessions (10–30 daily sessions), the minutes of exposure (2–3 min) and the temperature (−160 °C–−110 °C). After the WBC session, patients underwent different types of physical individual or group exercise (15–30 min), and sometimes this information was missing.

### 3.4. Outcome Measures

The parameters evaluated in the different studies varied from haematological tests to functional evaluations and an account of the psychological effects.

#### 3.4.1. Blood Values

Several studies on the effects of WBC on blood parameters have been performed by Ptaszek and colleagues [52,53,54,56]. Their overall results showed that there were no significant changes in the blood values after a cycle of 20 sessions of WBC (−120 °C/3 min), except for a statistically significant decrease in transferrin levels in MS patients [54], and a significant increase in erythrocyte deformability and a decrease in haematocrit [56].

Similar results were found by Bryczkowska et al., except for a rise in superoxide dismutase (SOD) activity, combined with a tendency for increased glutathione transferase (GST) activity in patients with MS [59]. Miller et al., in a series of studies, showed an increase in total antioxidant status (TAS) [48,61,62] and uric acid (UA) [60] after WBC treatment.

#### 3.4.2. Functional Evaluation

Several studies have evaluated functional tests after WBC treatment. In his study, Pawik found a statistically significant improvement in functional status using the Rivermead Mobility Index (RMI) [58]. In addition, Miller et al. found that WBC appeared to be effective in improving functional status, as measured by the RMA and the Multiple Sclerosis Impact Scale Physical Status (MSIS-29-PHYS), and fatigue, as measured by the Fatigue Severity Scale (FSS), in people with MS [41]. Another study by Radecka et al. showed that after WBC there was an increase in the amplitude of the extensor carpi radialis (ECR) and a decrease in the amplitude of the flexor carpi radialis (FCR), with significant differences in the resting sEMG signals between the ECR and the FCR. They also found an increase in handgrip strength (HGS), an improvement in gait, and a decrease in fatigue [55]. On the other hand, Lubkowska et al. only found an improvement in the strength of the right thumb [57]. A positive pre-post effect on the EDSS was also found by Miller et al. in two different studies [41,60].

#### 3.4.3. Psychological Effects

The psychological aspect of WBC treatment was also considered. An improvement on the Multiple Sclerosis Impact Scale psychological status (MSIS-29-PSYCH) after WBC was also found by Miller et al. [41]. Pawik found a significant difference on the Psychological General Well Being Index (PGWBI) in the groups of patients who underwent exercise alone or in combination with WBC, a significant reduction in the severity of anxiety symptoms measured by the Hospital Anxiety and Depression Scale (HADS-A) in the WBC + exercise group, and a significant reduction in depressive symptoms in the WBC groups [58].

### 3.5. Adverse Effects

This comprehensive review of studies revealed no evidence of adverse effects associated with the different WBC intervention protocols

## 4. Discussion

This section summarises the results of the literature (between 2010 and 2023) on the effects of WBC on MS with the aim of providing a comprehensive and up-to-date overview of the latest findings in the field. This review discusses the effects of cold stimulation on a wide range of physiological (i.e., haematological, metabolic, and endocrinological), functional (fatigue, strength, pain, and motor performance), and psychological (anxiety, depression, and well-being) parameters.

### 4.1. Physiological Parameters

#### 4.1.1. Antioxidative Capacity and Antioxidant Enzymes

Oxidative stress emerges when the formation of free radicals and the capability of cells to remove them is unbalanced and may underlie, with different levels of importance, the beginning and/or development of numerous diseases (e.g., cancer, diabetes, metabolic disorders, atherosclerosis, and cardiovascular diseases) [63].

The antioxidant defensive system consists of enzymatic components like superoxide dismutase (SOD), catalase (CAT), glutathione peroxidase (GPx), and thioredoxin (Trx), as well as the non-enzymatic antioxidants [64]. Enzymatic antioxidants can catalyse ROS to protect cells. Non-enzymatic antioxidants can counteract the oxidative effect by enhancing the antioxidant enzymes or by directly intervening in the oxidative chain reaction [64].

The first work by Miller et al. [62] compared changes in the total antioxidative status (TAS) and the SOD and CAT activities in the erythrocytes of MS patients, which are indicators of the body’s overall antioxidant status capacity, before and after a series of 10 WBCs (2–3 min/−120° to −110 °C) with a MS control group. Both groups received 30 min of physiotherapy consisting of progressive balance and strength exercises. The baseline level of the TAS in the MS patients was significantly lower than in the healthy subjects but increased after two weeks of WBC treatment compared with the MS control group (without WBC). Similarly, in the erythrocytes of MS patients, the basal level of SOD activity was significantly lower compared to the healthy group. However, its activity after the WBC treatment was not statistically altered. In contrast to the SOD activity, the CAT activity was 2-fold higher in the erythrocytes of MS patients than in healthy people, and exercise showed no impact on its action.

The TAS levels in MS patients presented an increase in oxidative stress, which was higher than in the healthy subjects and was reduced in both the group that only exercised and the group that participated in kinesiotherapy and received WBC treatment. This indicates that WBC causes an increase in TAS, similar to what was previously observed after physical exercise when ROS are produced [65], and could have an impact on the activation process of antioxidative properties in MS patients. However, the SOD and CAT activities remained unchanged, suggesting the positive antioxidant effects of WBC as a short-term complementary approach in MS.

Another work by the same group [61] confirmed that the plasma TAS was significantly lower in patients with SPMS than in the healthy group, indicating that the impaired antioxidant defence system in MS may depend in part on the reduced activity of SOD. The results also showed that after WBC, as well as after exercises in MS patients, the TAS was increased compared to untreated patients, but had no effect on anti-oxidative enzyme activity: the SOD and CAT. In addition, the authors noted a significant elevation in SOD and CAT activity in the RBCs of MS patients after the supplementation of melatonin with WBC, but the effects cannot be attributed to either melatonin or WBC, due to the lack of a proper control group (those with only melatonin were not exposed to WBC) [61]. The reduction in oxidative stress was accompanied by improvements in symptoms, including a clinically significant reduction in fatigue, an increase in muscle strength, and a reduction in disability.

The next study by Miller et al. [48] was conducted in patients with depressive and non-depressive MS and had the same aim as the previous two: to compare the effect of WBC on the plasma TAS and the activity of selected anti-oxidant enzymes in the erythrocytes (the SOD and CAT). The patients underwent ten WBC sessions which caused a significant increase in the TAS levels, reaching the values of the healthy controls, but showed no effect on the activity of the SOD and CAT. The data demonstrated that the plasma TAS level was lower in depressive MS patients than in the healthy group, once again indicating the impaired antioxidant defences in MS patients.

The study by Bryczkowska et al. [59] also confirms the possibility of the modulating effect of WBC on the systemic antioxidant potential, which can be considered as one of the factors leading to an improvement in the functional state of patients. In this study, as the series of treatments increased to 30 (3 min/−130 °C), only a significant increase in the SOD1 activity was observed, accompanied by a small upward trend in the GST activity and a downward trend in glutathione reductase and, unexpectedly, CAT.

Two studies by Ptaszek et al. [52,53] sought to assess the impact of a program of 20 WBC sessions on CAT, GPx, SOD, changes in TAS/TAC, and in the total oxidative status/total oxidative capacity (TOS/TOC) concentration in women with MS, finding no significant changes at the end of the experimental protocol between the study group and the control groups. In both studies, the temperatures inside the cryochamber were the same (−120 °C) as the incremental time of a single WBC treatment: 90 s (1° treatment), 120 s (2° treatments), and 180 s (3–20 treatments). Interestingly, there were also no significant baseline changes between the study groups as seen in the aforementioned studies. Therefore, these findings do not provide clear evidence to support the previous results in this area, possibly due to the differences in the interventions (20 vs. 10 WBC sessions and a warm-up on a cycle ergometer without resistance for 15 min versus 20–30 min of progressive balance and muscle strengthening exercises) [48,59,60,61,62].

In summary, the following reports were made about the WBC treatment:

- WBC can increase the TAS and could play an important role in the activation process of antioxidative properties in MS patients, suggesting the positive antioxidant effects of WBC as a short-term adjuvant treatment for MS.
- No significant changes in antioxidant enzymes (CAT, GPx, and R-GSSG) were detected, except for SOD when the WBC sessions increased to 30, suggesting that different cooling interventions could affect changes in the antioxidant capacity.

#### 4.1.2. Nitric Oxide

There is strong evidence that NO is involved in several features of MS (e.g., oligodendrocyte damage, demyelination, and axonal degeneration) and that it may contribute to a functional loss by affecting the axonal transmission [66,67,68]. As a consequence of inflammation, the inflammatory cells generate high levels of NO, which interferes with the electron transport in the mitochondria, blocking oxidative phosphorylation [69]. This mechanism causes hypoxia in the neuronal cells in inflammatory lesions. Elevated serum NO levels have also been reported to be related to the type of MS and relapse rate and may also reflect a physiologic reaction to generalised oxidative stress [70]. Moreover, elevated concentrations of NO have also been found in the urine and cerebrospinal fluid of MS patients [71,72].

Although there is still some controversy on this topic, most studies on the involvement of NO in MS report higher NO activity in MS patients compared to the controls [73,74]. Cooling demyelinated nerves with cooling suit therapy can reduce the conduction block through a decrease in NO concentrations, improving the symptoms of MS [75,76]. However, the study of Ptaszek et al. reported no significant impact of WBC on NO concentrations but just a downward trend after WBC in MS patients [53].

In summary, the following reports were made about the WBC treatment:

- No significant impact of WBC on NO concentration was reported.

#### 4.1.3. Uric Acid Plasma Concentration

Uric acid (UA) is the end product of purine metabolism and is the major endogenous non-enzymatic antioxidant in the human body, accounting for over half of the plasma antioxidant capacity [77]. Hooper et al. were the first to document that patients with MS had a significant lower serum uric acid level than controls with spinal cord injury or Parkinson’s disease [78]. In addition, they found that MS and gout (hyperuricemia) are almost mutually exclusive diseases, raising the possibility that high UA concentrations (hyperuricemia), as a potent antioxidant, may protect against MS, reducing the risk of MS development or decreasing its progression, whereas the decreased UA levels in MS indicate a primary constitutive reduction in protection against oxidative stress [79].

However, results on the correlation of its concentration with disease progression, disease MRI activity, disease subtype, disease disability, or MS risk prediction are poor or mixed [80]. Thus, a neuroprotective potential of UA has been speculated, and the serum level of UA may be a potential biomarker for disability associated with MS and for neuromyelitis optica [53].

The long-term effects of WBC on UA concentrations in MS patients were firstly observed by the group of Miller et al. [60]. WBC increased plasma UA levels in patients with SPMS not only immediately, but also one and three months later. Moreover, WBC induced positive changes in the EDSS perceptual scale immediately after WBC, which were maintained 1 and 3 months later. Although the investigators did not observe a statistically significant correlation between the plasma concentration of UA and the degree of disability in people with MS, they did observe a trend towards a negative correlation.

Furthermore, the study by Ptaszek et al. showed lower baseline UA concentrations in women with MS in the study group and in the control group, although these were not statistically significant [53]. In addition, there were no statistically significant changes in the UA levels after 20 WBC sessions.

In summary, the following reports were made about the WBC treatment:

- WBC can increase the plasma UA levels in SPMS patients not only immediately, but also one and three months later.

#### 4.1.4. Metalloproteinases’ Serum Levels

Metalloproteinases (MMPs) are a highly homologous, multidomain, big family of zinc (Zn^2+^)-containing endopeptidases that degenerate various proteins of the extracellular matrix and the basement membrane, such as collagens, gelatin, laminin, and fibronectin [81]. Based on their ability to degenerate basal lamina components and their involvement in damage to the blood–brain barrier (BBB), the role of MMPs, especially MMP-9 and MMP-2, has been widely studied in the brain tissue, serum, and cerebrospinal fluid of MS patients [82]. Interestingly, the circulating levels of MMP-9 have been shown to be upregulated in MS patients, in contrast to other non-inflammatory neurological disorders and healthy subjects [83,84]. Serum MMP-9 has typically been observed in patients with an active disease, which is manifested by a continuous clinical relapse [83]. Similarly, the results of the cerebrospinal fluid MMP-9 levels also increased among individuals during the active disease and with active RRMS patients [85,86]. Moreover, higher plasma MMP-9 levels are related to the severity of the disease [87].

The only study addressing the levels of MMP-9 obtained the contrary results: higher significant baseline levels in healthy women were compared with the control group with MS and to the study group with MS, although they were statistically insignificant. WBC also had no effect on the MMP-9 serum concentration in females with MS, while a decrease (that was not significant) was observed in healthy women after WBC [53].

In summary, the following reports were made about the WBC treatment:

- WBC seems to have no effect on the level of MMP-9 in females with MS.

#### 4.1.5. Haematological Parameters and Inflammatory Markers

Iron participates in a multitude of bodily processes and is essential for oxidative phosphorylation and myelin formation [88]. An excess of the free ion can be toxic and its high deposition in tissues is associated, among many chronic diseases, with MS, where its accumulation in the CNS leads to neurotoxicity through mechanisms that include oxidative stress, glutamate excitotoxicity, protein misfolding, and ferroptosis [89]. However, the serum levels of transferrin and ferritin are not always altered in MS, as studies showing alterations contrast with others showing no difference in concentrations [90], such as the study by Ptaszek et al. who reported no change in the mean values of transferrin, ferritin, and iron in people with MS compared to healthy people, but only a significantly lower level of transferrin after 20 sessions of WBC, although within normal limits. This change could be related to the 15 min warm-up on a cycle ergometer that the patients performed after WBC [54].

Some trophic factors have been shown to enhance the survival of cells and to have a protective effect against neuronal degeneration: Brain-derived Neurotrophic Factor (BDNF), Nerve growth factor (NGF), Platelet-derived growth factor (PDGF), Vascular Endothelial Growth Factor (VEGF), and Insulin Growth Factor I (IGF-I) [91,92].

BDNF plays an active part in the processes of neuroregeneration, neuroprotection, and neurogenesis, and lower levels can accelerate the onset of disability [93]. NGF is essential for the differentiation, regeneration, and growth of neurons in the peripheral nervous system and for the correct function of cholinergic neurons in the CNS [94]. PDGF is one of the most significant contributors to MS remission, stimulating neuronal differentiation, remyelination, and an increased density of oligodendrocytes [95]. IGF-I may play a fundamental role in the myelination process [96].

Their increased levels are expected to correlate with improved neuroplasticity in MS. However, the changes observed by Ptaszek et al. were not statistically significant, either at baseline or after WBC [54].

Despite MS being characterised as a chronic inflammatory disease of the CNS, Ptaszek et al. observed no changes in the levels of IgG, IgA, IgM, and C-reactive protein levels (CRP), although studies on the latter are inconsistent regarding its association with MS [54].

Several conditions can be diagnosed if the haematological parameters are outside the health-related reference interval. Nevertheless, haematological changes in MS patients are not characteristic. Decreased hemoglobin levels, probably associated with enhanced hemolysis, is a specific but temporary feature of the WBC that may simply reflect a temporary physiological adaptation [97,98]. Furthermore, haematological changes could be influenced by the number of WBC sessions, and a temporary decrease in Haemoglobin (HGB), Haematocrit (Hct), and Red Blood Cells (RBCs) after 10 and 20 sessions or an increase after the 30 sessions is possibly due to a recovery in erythropoiesis [99].

Given the scarcity of studies, Ptaszek et al. studied the effects of 20 WBC sessions on biochemical and rheological blood indices in patients with MS [56]. They found no significant effect on changes in blood counts in patients with MS compared to the control group. However, the patients with MS had statistically significant lower levels of RBC and HGB at baseline compared to healthy women. In addition, a slight reduction in mean corpuscular volume (MCV) and HGB and a statistically significant minimal increase in mean corpuscular haemoglobin concentration (MCHC) was observed in healthy women after WBC.

Moreover, after WBC, the authors observed a significantly reduced Hct value (within the physiological range) in the healthy women, which may be responsible for the increase in the deformation capacity of the erythrocytes, which positively influenced the rheological properties of blood, since HcT is important for the flow of blood cells through capillaries with a diameter two times smaller than their own [100].

In clinical practice, plasma fibrinogen, whose levels increase two- to three-fold during the inflammatory response that causes cell aggregation, may be an important and easily detectable biomarker of activity during a relapse in MS patients [101]. However, women with MS have not been found to have elevated fibrinogen levels. Only a statistically significant increase in the fibrinogen concentration was detected after 20 WBC treatments in healthy women, but larger groups are needed to confirm these results, given the wide variation in people with MS.

Blood platelets also play an important role in the coagulation cascade and are found in large numbers in MS lesions [102], but, in the same study by Ptaszek et al., no statistically significant changes were found in the PLTs after WBC use or in the basal levels [56].

In summary:

- A cycle of 20 WBCs did not influence changes in the blood counts, rheology, and biochemistry in women with MS.
- The use of WBC had a positive effect on the rheological properties of blood, increasing the deformability of erythrocytes and decreasing the Hct values (within the physiological norms) of healthy women.
- WBC does not adversely affect RBC deformability and aggregation, making it safe for MS patients.

### 4.2. Physical, Functional, and Psychological Parameters

MS causes a range of symptoms that can interfere with daily activities and needs to be addressed by being treated or managed [3,60]. The progressive forms of MS are characterised not only by a sustained accumulation of disability but also by a worsening of cognitive impairment and fatigue, which may be associated with brain atrophy and neurodegenerative processes [103].

As symptoms progress, conventional therapy becomes ineffective and new therapeutic approaches that are targeted at enhancing the patients’ functional status and quality of life are required [104].

It has been shown that up to 60–80% of people with MS experience a worsening of their symptoms when they are subjected to higher ambient temperatures and/or increased core body temperatures, such as during exercise (Uhthoff’s phenomenon) [105,106]. On the other hand, several studies showed that cooling can enhance the motor abilities of MS patients and may improve some MS symptoms [41,60,107,108,109].

Some research groups investigated the effect of WBC on physical, functional, and psychological effects in patients with MS. In their 2013 study, Miller et al. observed a longitudinal improvement in the functional status of SPMS patients, as measured by the EDSS scale, not only immediately after a cycle of 10 WBC treatments (3 min/−130 °C) but also at a 3-month follow-up [60]. The authors claimed that the results obtained could be associated with improvements in fatigue and cognitive status and with antioxidant effects.

In 2016, the same group investigated the effect of 10 WBC sessions (2–3 min, at −110°–−160 °C) on fatigue, disability, and the physical, functional, and psychological status in two groups of patients with MS with different levels of fatigue [41]. The results showed significant improvements in functional and psychological status in both groups, particularly in the “high-fatigue” group, due to the significant reduction in fatigue observed in both groups. These results suggest that WBC may be a very helpful treatment for all people with MS, especially when fatigue appears as a highly disabling factor reducing the patient’s autonomy and performance.

Another research group analysed the effects of 10 WBC treatments (−110°/−160 °C), randomly dividing patients into three groups: patients who had WBC combined with physical exercise training (WBC–Gym group), patients who only had WBC treatments (WBC group) and the last who only had exercise training (Gym group) [58]. The results showed that WBC–Gym mainly improved psychological and general well-being, evidencing that cryostimulation has an important influence on mental and physical health, particularly in improving patients’ mental state.

These findings are consistent with previous studies conducted by Rymaszewska investigating the positive effects of cryostimulation on psychological well-being, suggesting that WBC (2–3 min, from –160 °C to –110 °C) may be beneficial in the treatment of patients with affective and anxiety disorders [110]. In addition, in 2020 the same group conducted a randomised controlled trial in which patients exposed to ten 2 min sessions of WBC (from −110 °C to −160 °C) showed significant improvements in depressive symptoms, quality of life, mood, and disease acceptance compared to the control group (WBC at −50 °C, considered as “non-cryogenic”), confirming that WBC could be an effective and safe intervention in patients with depressive disorders [43]. However, the exact mechanisms explaining the beneficial effect of WBC on mood disorders remain unresolved, with speculation that possible mechanisms are linked to modulation of the overall inflammatory response [43].

Furthermore, a study by Radecka et al., which was the first to investigate the potential influence of WBC on muscle activity in patients with MS, showed an improvement in the functional status of MS patients, with a reduction in fatigue and an increase in grip strength and walking speed after 20 WBC exposures (2–3 min, at −110 °C) compared to the control group [55]. The improvement in functional status and the decreased fatigue in MS patients after daily sessions of WBC might be ascribed to adaptive changes in bioelectrical muscle activity, although the lack of studies on the subject makes further research necessary.

In summary:

- WBC can lead to a reduction in fatigue and an improvement in functional status, potentially improving the overall well-being and quality of life of people with MS.
- Cryostimulation has a significant effect on both mental and physical well-being, with a particular focus on improving the symptoms of mood, anxiety, and depression.
- The effects of WBC on improvements in functional status observed in people with MS may be due to adaptive changes in bioelectrical muscle activity.

### 4.3. Limitations

There are several limitations to this scoping review. There is a lack of insight into the molecular mechanisms involved in the observed responses to treatment, which also explains the higher number of studies investigating the variation in haematological parameters in MS after WBC. The heterogeneity of the population, different types of MS, and the lack of WBC standardised protocols (i.e., temperature, number of sessions, and duration of exposure) among the studies should be considered. The different outcome measures and the limited data reported did not allow a proper comparison between studies or a systematic review. Furthermore, although this study adopted a non-systematic approach, an alternative meta-analysis would not have provided additional information given the type and quality of the available articles. Therefore, given the small number of studies published on this topic, the authors preferred to conduct a scoping review.

## 5. Conclusions

The therapeutic approach in MS is still challenging due to the variety of overlapping symptoms limiting the possibilities of pharmacological treatment. Therefore, in addition to pharmacotherapy, add-on treatment methods are still needed to help reduce the disability, and, in this regard, physiotherapy and physical activity yield an established, important role.

However, an emerging complementary treatment such as WBC promises to increase the efficacy of such interventions, partly and especially because it has been shown to be safe, acceptable, and with a low risk of side effects, since adverse events appear to be rare in relation to the extent of WBC growth worldwide [39,42,111].

The data analysed in this review suggest that WBC could complement the therapeutic armoire for MS patients, since the effects of cryogenic cold stimulation have been shown to activate antioxidative processes with consequent improvements in functional status, mood, anxiety, and fatigue.

Despite the limitations of the available studies, it appears that WBC can reduce MS symptoms and enhance the effects of rehabilitation programs, which is interesting in terms of the cost-effectiveness of rehabilitation. Last but not least, the high compliance and satisfaction with the treatment and the absence of side effects reported in most of the studies seem to indicate that WBC is a component of the rehabilitation programme, which is crucial for the long-term management of MS.

## 6. Future Perspectives

The studies included in this review provided initial evidence of the effectiveness of WBC for the treatment of MS. However, in view of the limitations of the included studies, this review article cannot draw final conclusions about the effectiveness of WBC in MS. The available data are still scarce, and the quality of the studies considered is not sufficient to generalise the results. Further studies are needed to determine the optimal number of WBC sessions, the temperature, the duration of cryogenic exposure in relation to individual physiological and anthropometric profiles, and the length of anti-inflammatory, antioxidant, and metabolic actions induced by a series of WBC sessions. It is also important to identify which MS population (relapsing or progressive) and which symptoms (e.g., fatigue and spasticity) would mainly benefit from integration of WBC into a multidisciplinary approach.

## Figures and Tables

**Table 1:** PICOS Criteria.

Criteria	Application
Population	Patients with MS
Intervention	WBC
Comparisons	Comparing before and post intervention
Outcomes	Technique-related improvements
Study design	Clinical trial, prospective trial, observational study

**Table 2:** Search Strategy.

Database	Query Terms
PubMed	(“whole-body cryostimulation” OR “whole-body cryotherapy”) AND multiple sclerosis
ScienceDirect	(“whole-body cryostimulation” OR “whole-body cryotherapy”) AND “multiple sclerosis”
Web of Science	((ALL = (“whole-body cryostimulation”)) OR ALL = (“whole-body cryotherapy”)) AND ALL = (“multiple sclerosis”)
Embase	(“multiple sclerosis”/exp OR “multiple sclerosis” OR “ms”) AND (“whole body cryotherapy”/exp OR “whole body cryotherapy” OR “whole body cryostimulation”/exp OR “whole body cryostimulation”)

**Table 3:** Data extracted from the included studies.

Author, Year	Study Design	Patients’ Features	WBC Application Modality	Outcome Measures	Results	Adverse Effects
Ptaszek et al., 2023 (a) [52]	Prospective controlled trial EG: MS vs. CG: healthy subjects	30 (EG: 15 vs. CG: 15) Age: EG: 41.53 vs. CG: 38,47 Sex: EG: 15F vs. CG: 15F EG: EDSS: 3.03	Wroclaw-type liquid nitrogen-cooled cryochamber, 20 daily WBC sessions 5/week (3 min, at −120 °C) After each treatment, there was a 15 min warm-up on a cycle ergometer.	CAT, GPx, and SOD. Before the start of treatment (T0) and the end of the series (T1).	No changes in the examined parameters (CAT, GPx, and SOD) in the MS or control groups.	Not reported
Ptaszek et al., 2023 (b) [53]	Prospective controlled trial EG: WBC—MS vs. CG1: No WBC—MS vs. CG2: Healthy—WBC	50 F (EG: 15 vs. CG1: 20 vs. CG2: 15) Age: EG: 41.53 vs. CG1: 40.45 vs. CG2: 38.47 EDSS: EG:3.03 vs. CG1:3.08	Wroclaw-type liquid nitrogen-cooled cryochamber, 20 daily WBC sessions 5/week (3 min, at −120 °C) After each treatment, there was a 15 min warm-up on a cycle ergometer.	TAS/TAC, TOS/TOC, NO, UA, and MMP-9. Before the start of treatment (T0) and at the end of the series (T1). There was a one-time (at baseline) examination of women without WBC intervention.	An insignificant increase in total antioxidant capacity and an insignificant decrease in total oxidative status/total oxidative capacity were observed in the EG. In women with MS, there was no significant effect of cryotherapy on changes in the oxidant/antioxidant imbalance or the concentrations of NO or MMP9.	Not reported
Ptaszek et al., 2022 [54]	Prospective study (MS vs. HC)	30 MS: 15 vs. HC: 15 Age: MS: 41.53 vs. HC: 38.47 Sex: MS: 15 vs. HC: 15 EDDS: 3.03	Liquid nitrogen-cooled cryochamber, 20 daily WBC sessions 5/week (first treatment, 1.5 min; second treatment, 2 min; and 3–20 treatments, 3 min) at −120 °C. After each treatment, there was a 15 min warm-up on a cycle ergometer.	Iron, transferrin, ferritin serum concentrations, IgG, IgA, IgM, CRP, BDNF, NGF, PDGF, VEGF, and IGF-1. Blood samples were collected before the start of the WBC procedures and after a series of 20 WBC sessions.	A statistically significant decrease in transferrin levels was observed in MS patients (within normal limits—this change could be related to the exercises patients did after WBC or to inflammation). When the other indicators were analysed, favourable directions and trends of changes were observed. However, the changes were not statistically significant.	Not reported
Radecka et al., 2021 [55]	RCT single-blind (EG: WBC vs. CG: no WBC)	114 (EG: 60 vs. CG: 54) Sex: EG: 44 F vs. CG: 39 F Age: EG: 44.95 vs. CG: 45.09 EDSS: EG: 1.76 vs. CG: 1.79	20 daily WBC sessions 5/week (2–3 min, at −110 °C). There was a 15 min group kinesiotherapy session after each treatment.	FSS, T25-FW, HGS, and sEMG of the ECR and FCR muscles of the wrist, for the dominant hand. Before the start of treatment (T0), between the second and fourth day, and at the end of the series (T1).	In EG, there was an increase in ECR and a decrease in FCR amplitude, with significant differences in resting sEMG signals between ECR and FC, an increase in HGS, gait improvement, and a decrease in fatigue. No significant changes were observed in CG.	Not reported
Ptaszek et al., 2021 [56]	Prospective controlled trial 3 arms EG: WBC—MS CG1: NO WBC—MS CG2: Healthy—WBC	50 F (EG: 15 vs. CG1: 20 vs. CG2: 15) Age: EG: 41.53 vs. CG1: 40.45 vs. CG2: 38.47 EDSS: EG:3.03 vs. CG1:3.08	Wroclaw-type liquid nitrogen-cooled cryochamber, 20 daily WBC sessions 5/week (first treatment, 1.5 min; second treatment, 2 min; and 3–20 treatments, 3 min) at −120 °C. After each treatment, there was a 15 min warm-up on a cycle ergometer.	White blood cells, RBC, Hb, HCT, PLT, MCH, MCV, MCHC, RBC, Elongation and Aggregation Indexes, protein serum level, and fibrinogen concentration. Before the start of treatment (T0) and at the end of the series (T1).	There was no significant effect on changes in blood counts, rheology, and biochemistry. WBC significantly increased the deformation capacity of erythrocytes and reduced the hematocrit value (within physiological norms) of healthy women, which had a positive effect on the rheological properties of blood.	Not reported
Lubkowska et al., 2020 [57]	Clinical trial WBC—MS	25 WBC—MS Age: 44.58 Sex: 19 F EDDS: ≤6	20 daily WBC sessions 5/week (2–3 min, at −110 °C). After each treatment, there was a 30 min session of individual physical rehabilitation.	HGT, T25-FW, and TS. Before the start of treatment (T0) and at the end of the series (T1).	A marginal but statistically significant increase in thumb strength was observed in the right hand. Other changes in HGT, TS, and T25-FW were not statistically significant.	Not reported
Pawik et al., 2019 [58]	Clinical trial WBC + GYM vs. WBC vs. GYM	60 (WBC + GYM: 20 vs. WBC: 20 vs. GYM: 20) Sex: WBC + GYM: 17 F, 3 M vs. WBC: 11 F, 8 M vs. GYM: 15 F, 5 M Age: WBC + GYM: 48.8 vs. WBC: 45.8 vs. GYM: 53.3 EDDS: WBC + GYM: 2.4 vs. WBC: 2.3 vs. GYM: 2.35	10 daily WBC sessions, 5/week (at −110°/−160 °C).	PGWBI, HADS, and RMI. Assessments were made before treatment, 14 days after the start of treatment, and 2 days after the end of the last session.	A statistically significant difference in PGWBI was found in the GYM and WBC + GYM groups. A statistically significant reduction in the severity of anxiety symptoms (HADS-A) was seen in the WBC + GYM group. A significant reduction in depressive symptoms (HADS-D) was seen in the WBC + GYM and WBC groups. A statistically significant improvement in functional status (RMI) was seen in the WBC group.	Not reported
Bryczkowska et al., 2018 [59]	Observational study	30 F Age 45.6 EDDS: >6	Wroclaw-type liquid nitrogen exchanger cryochamber, 30 daily WBC sessions (3 min, at −130 °C). There was a 30 min group kinesiotherapy session after each treatment.	SOD, CAT, GPx, R-GSSG, GST, total protein, albumin, glucose, and uric acid levels; and Tch, HDL, LDL, and TAG concentrations.	No significant changes in total protein, albumin, uric acid, glucose concentrations, total cholesterol, HDL and LDL cholesterol levels, and triacylglycerol concentrations were observed, and a significant increase in SOD1 activity was associated with a trend towards increased GST activity.	Not reported
Miller et al., 2016 [41]	Case-control study 2 groups LF (FSS 38–42) vs. HF (FSS 48–52)	48 (LF: 24 vs. HF: 24) Sex: (LF: 14 F vs. HF: 14 F) Age LF: 55.6 vs. HF: 55.7 EDDS LF: 5.2 vs. HF: 5.1	KR2005N-type liquid nitrogen-cooled cryochamber, 10 daily WBC sessions, 5/week, (2–3 min, at −110°–−160 °C).	RMA (RMA 1 gross function, RMA 2 leg and trunk, RMA 3 arm), MSIS-29 (MSIS-29 PHYS, MSIS-29-PSYCH), and EDSS. Before the start of treatment (T0) and at the end of the series (T1).	In both groups, there was a significant improvement in functional status and fatigue, with the changes observed in HF patients being significantly greater, especially in the MSIS-29-PHYS, MSIS-29-PSYCH, RMA1, and RMA3. WBC appears to be effective in improving functional status and fatigue in people with MS, especially in those who are most fatigued.	Not reported
Miller et al., 2013 [60]	Clinical trial EG: SPMS vs. CG: healthy	44 (EG: 22 vs. CG: 22) Sex: EG: 12 F vs. CG: 12 F Age: EG: 48.6 vs. CG: 45.8 EG: EDSS: 4.5	Liquid nitrogen-cooled cryochamber, 10 daily WBC sessions, 5/week, (3 min, at −130 °C).	UA plasma concentration, EDSS. Four-stage examination: before the WBC (0), directly after 10 days of WBCT (I), and 1 month (II) and 3 months (III) after completion of the series.	WBC increased plasma UA levels in SPMS patients not only immediately, but also one and three months later. In addition, WBC induced positive changes in the EDSS scale both immediately and after 1 and 3 months.	Not reported
Miller et al., 2011 [48]	Clinical trial MS-D WBC vs. MS non depression WBC	22 (15 F) Age: 42.2 (MS-D: 12 vs. MS—non-D: 10) EDSS: 4.5	10 daily WBC sessions in a liquid nitrogen-cooled cryochamber, from Monday to Friday (2–3 min at −110°/−160 °C).	TAS, SOD, and CAT. 1h before the start of treatment (T0) and 1h after the end (T1).	WBC increased the level of TAS in depressive MS patients more than in non-depressive MS patients. WBC treatment resulted in a significant increase in plasma TAS levels but had no effect on the activities of SOD and CAT.	Not reported
Miller et al., 2010 (b) [61]	Clinical trial MS—WBC vs. HC	32 MS—WBC: 16 vs. HC: 16 Age: MS—WBC: 43.2 Sex: MS—WBC: 11F MS—WBC: EDSS: 4.5	MS—WBC: 3 cycles of 10 daily WBC sessions in a liquid nitrogen-cooled cryochamber, from Monday to Friday (2–3 min at −110°/−160 °C). HC: 1 cycle of 10 daily WBC sessions in a liquid nitrogen-cooled cryochamber, from Monday to Friday (2–3 min at −110°/−160 °C).	TAS, SOD, and CAT. MS-WBC: patients were examined at two stages at the beginning of the third cycle of WBC treatment. HC: patients were examined at two stages at the beginning of the end of WBC treatment.	WBC increased TAS but had no effect on the activity of antioxidant enzymes: SOD and CAT.	Not reported
Miller et al., 2010 (a) [62]	Clinical trial MS—WBC vs. MS—no WBC vs. HC	52 MS—WBC: 16 vs. MS—no WBC: 16 vs. HC: 20 Age: not specified Sex: not specified EDDS: not specified	10 daily WBC sessions in a liquid nitrogen-cooled cryochamber, from Monday to Friday (2–3 min at −110°/−160 °C).	TAS, SOD, and CAT. 1h before the start of treatment (T0) and 1h after the end (T1) MS patients were assessed at the beginning and end of treatment. HC had one examination.	The level of TAS in MS patients was significantly reduced compared to HC. A significant increase in TAS was observed in the WBC group compared to the CG.	Not reported

BDNF, Brain-derived neurotrophic factor; CAT, Catalase; CG: control group; CRP, C-Reactive Protein; ECR, extensor carpi radialis; EDSS, Expanded Disability Status Scale; EG: experimental group; FCR, flexor carpi radialis; FSS, Fatigue Severity Scale; GPx, glutathione peroxidase; GST, glutathione transferase; HADS, Hospital Anxiety and Depression Scale; Hb, Haemoglobin; HC: healthy control; HCT, hematocrit; HDL, high density lipoprotein; HF, high fatigue; HGS, hand grip strength; IGF-1, Insulin-like growth factor-1; LDL, low-density lipoprotein; LF, low fatigue; MCH, Mean Corpuscular Haemoglobin; MCHC, Mean corpuscular haemoglobin concentration; MMP-9, Matrix metalloproteinase-9; MS, Multiple Sclerosis; MS-D, Multiple Sclerosis depression; MSIS-29, Multiple Sclerosis Impact Scale; MSIS-29-PHYS, Multiple Sclerosis Impact Scale physical status; MSIS-29-PSYCH, Multiple Sclerosis Impact Scale psychological status; NGF, Nerve growth factor; NO, Nitric oxide; PDGF, Platelet-derived growth factor; PGWBI, Psychological General Well Being Index; PLT, Platelet Count; RBCs, Red Blood Cells; R-GSSG, glutathione reductase; RMA, Rivermead Motor Assessment; RMI, Rivermead Mobility Index; sEMG; surface electromyography; SPMS, Secondary Progressive Multiple Sclerosis; SOD; superoxide dismutase; T25-FW, Timed 25-Foot Walk; TAG, triacylglycerol; TAS, total antioxidative status; TAS/TAC, total antioxidant status/total antioxidant capacity; Tch, total cholesterol; TOS/TOC, total oxidative status/total oxidative capacity; TS, thumb grip strength; UA, Uric Acid; VEGF, Vascular endothelial growth factor; and WBC, Whole-body cryostimulation.

## Data Availability

The data presented in this study are available within this article.
